# Adipose stem cell-derived exosomes in the treatment of wound healing in preclinical animal models: a meta-analysis

**DOI:** 10.1093/burnst/tkae025

**Published:** 2024-08-04

**Authors:** Jing-tao Wei, Ting He, Kuo Shen, Zhi-gang Xu, Jun-tao Han, Xue-kang Yang

**Affiliations:** Department of Burns and Cutaneous Surgery, Burn Center of PLA, The First Affiliated Hospital of Air Force Medical University, Chang-Le Xi Street#127, Xi'an 710032, China; Department of Burns and Cutaneous Surgery, Burn Center of PLA, The First Affiliated Hospital of Air Force Medical University, Chang-Le Xi Street#127, Xi'an 710032, China; Department of Burns and Cutaneous Surgery, Burn Center of PLA, The First Affiliated Hospital of Air Force Medical University, Chang-Le Xi Street#127, Xi'an 710032, China; Department of Burns and Cutaneous Surgery, Burn Center of PLA, The First Affiliated Hospital of Air Force Medical University, Chang-Le Xi Street#127, Xi'an 710032, China; Department of Burns and Cutaneous Surgery, Burn Center of PLA, The First Affiliated Hospital of Air Force Medical University, Chang-Le Xi Street#127, Xi'an 710032, China; Department of Burns and Cutaneous Surgery, Burn Center of PLA, The First Affiliated Hospital of Air Force Medical University, Chang-Le Xi Street#127, Xi'an 710032, China

**Keywords:** Adipose stem cell, Exosomes, Wound healing, Preclinical study, Meta-analysis, Neovascularization, Epithelization, Scar

## Abstract

**Background:**

Wound healing has always been a serious issue for doctors and primary health care systems. In addition, adipose stem cell-derived exosomes have been proven to play a positive and effective role in tissue repair and regeneration. A systematic review of these preclinical studies was performed to assess the efficacy of adipose stem cell-derived exosomes (ADSC-Exos) in treating wounds. This article aimed to study the effectiveness of ADSC-Exos for the treatment of animal skin wounds and includes a meta-analysis of exosomes from general wounds and diabetic ulcer wounds in *in vitro* models of animals to provide a theoretical basis for clinical translation.

**Methods:**

A total of 19 studies with 356 animals were identified by searching the PubMed, Cochrane, MEDLINE Complete, Web of Science, CNKI and Wanfang databases from inception to 15 November 2022. No language or time restrictions were applied. Stata17 was used for all the data analyses.

**Results:**

The meta-analysis showed that ADSC-Exo therapy significantly improved the wound healing rate in the control group, except in the diabetes group on day 7. Day 7 of general wounds [standard mean difference (SMD) 2.87, 95% confidence interval (CI) 1.91–3.83)] and day 14 (SMD 2.89, 95%CI 1.47–4.30). Day 14 (SMD 3.43, 95%CI 1.28–5.58) of diabetic wounds. Other outcomes, such as blood vessel density, collagen deposition and wound re-epithelization, improved with the administration of ADSC-Exos.

**Conclusions:**

A meta-analysis showed that ADSC-Exo therapy applied to general and diabetic wounds can promote neovascularization, improve epithelization and collagen fiber deposition, promote healing, and reduce scar formation. ADSC-Exos have broad potential in preclinical research and clinical fields.

Highlights

- ADSC-Exos can promote the effective healing of common and diabetic wounds.
- A meta-analysis result showed that ADSC-Exos were used in animal experiments for skin wound repair.
- Compared with the control group, ADSC-Exo therapy can significantly improve wound healing rate.

## Background

The intact skin is an important protective barrier for the human body, and tissue injury and repair are continuous issues for doctors and primary health care systems. Wounds are usually divided into acute and chronic wounds. Acute wounds are often caused by external injury factors or surgery. Chronic wounds are often caused by comorbidities and underlying long-term conditions, such as diabetic foot ulcers, leg ulcers, foot ulcers, varicose eczema and bed sores. In particular, effective management of chronic wounds is a serious health care issue. Chronic wounds are prone to complications due to prolonged healing times, frequent dressing changes and improper management, such as ischemia or necrosis [1]. The adverse effects of chronic wounds are affecting an increasing number of families through increased psychological burden, reduced quality of life and increased medical expenses [2–4]. In Europe and the USA, the cost of treating wounds often exceeds tens of billions of dollars or pounds [4, 5]. If the wound is combined with complications such as diabetes, ulcers, infections or amputations, the cost will increase even more significantly, resulting in a substantial financial burden [6, 7].

In patients with chronic wounds, local and systemic factors such as advanced age, ischemia, infection, oxygenation, nutritional deficiency or diabetes can delay or prevent wound healing [5, 8]. In recent years, during the COVID-19 pandemic, the frequency of outpatient visits has been limited, resulting in more wounds not being treated promptly and effectively, which may not only prolong their healing time but also negatively impact wound outcomes [9, 10]. At present, various wound repair techniques, including surgical intervention, new dressings, negative pressure occlusion and drainage, skin substitutes, topical drugs etc., have achieved certain clinical effects; however, in patients with anti-inflammatory and reduced scar hyperplasia, these factors make it more challenging to achieve rapid and complete wound healing. With the increase in the number of diabetic wounds, the management of refractory wounds is usually performed by combination therapy. In previous studies, surgical debridement, vacuum sealing drainage, local topical drugs and traditional Chinese medicine were used to treat diabetic wounds. However, traditional wound treatment focuses on physical debridement and repair, while the degree of vascularization and inflammatory response of the wound affect the restoration of the microenvironment. Although surgical debridement removes severely polluted or inactive necrotic tissue, there are still limitations in active regulation during the process of wound healing. Multiple rounds of debridement can also cause long wound-healing times, poor healing quality, complications and other problems, thus increasing medical burden. As a noninvasive treatment, wound dressings have the advantages of easy preservation, convenient use and effective attraction to wound exudates, but they lack immune regulatory function. Therefore, the anoxic and humid microenvironment formed after an infected wound is covered by a dressing further increases the infection rate of the wound and cannot meet the needs of wound healing. Moreover, negative pressure closed drainage can block contact between the wound and the external environment, promote the growth of granulation tissue and drain the wound exudate, but the use of materials with negative pressure and different pressure levels is still controversial in clinical application [11].

In recent years, an increasing number of scholars have proven that mesenchymal stem cell (MSC) exosomes have great potential for wound healing and have broad clinical application prospects, not only for achieving scar-free wound healing with cell-free therapy in normal wounds but also for effectively treating ischemic wounds and diabetic wounds [12, 13]. The mechanism of action of exosomes has been proven through animal and cell experiments; exosomes can upregulate the expression of wound anti-inflammatory cytokines, promote the activation of macrophages, the multiplication and activation of fibroblasts, and the production of new blood vessels, and reduce scarring via a variety of mechanisms [14–18]. Compared with other pluripotent stem cells, fat-derived stem cell exosomes have the advantages of abundant sources, minimal adverse effects and immune tolerance [19, 20]. Adipose tissue can provide nearly 100 times more stem cells than bone tissue, unlike bone marrow, which is derived from bone marrow in very small quantities and requires puncture. Moreover, the ability of adipose-derived stem cells (ADSCs) to differentiate into endothelial cells is greater than that of bone marrow mesenchymal stem cells [21]. The source of exosomes from adipose stem cells is also better than that from endometrial mesenchymal stem cells, which are derived from endometrial tissue, exist only in women, have few sources and are inconvenient to obtain.

In previous studies, ADSC-exosomes (ADSC-Exos) were shown to have promising results in three overlapping phases of the skin regeneration process, namely, the inflammatory phase, the proliferative phase (which includes cell proliferation and regenerative epithelialization) and the remodeling phase. ADSC-Exos deliver bioactive substances such as mRNAs, microRNAs (miRNAs), DNAs, proteins, cytokines and growth factors through paracrine effects, thereby affecting cell function and providing a new therapeutic strategy for wound repair [22, 23]. Other studies have confirmed that the upregulation of epidermal growth factor and vascular endothelial growth factor can promote wound granulation tissue formation, vascular regeneration and re-epithelialization. Reducing the expression of inflammatory factors, promoting the polarization of M2 macrophages, reducing wound inflammation, promoting the formation of type I and type III collagen in fibroblasts at different stages of wound repair, or downregulating the expression of related miRNAs is conducive to accelerating wound healing and reducing scar hyperplasia [19, 24, 25]. According to relevant studies in recent years, ADSCs can be differentiated into various lineages and promote the repair and regeneration of myocardial, bone, nerve, skin and other tissues. ADSCs also have certain application prospects for treating cardiovascular and cerebrovascular diseases, lung injury due to sepsis, inflammatory bowel disease and specific dermatitis, and they improved androgenic alopecia and could be used in new strategies and methods for cell-free treatment. Other scholars have combined exosomes with biological materials such as gelatin methacryloyl and 3D-printed tissue-engineered skin substitutes to control the release of exosomes, and found that local high concentrations could be maintained at the wound site [26]. By enhancing activity and accelerating wound healing, new ideas for health management can be developed. In addition, studies in the field of ADSC-Exos have shown that they improve the recovery of cardiac function in mice and repair damaged myocardial tissue to a certain extent through the miRNA-205 signaling pathway [27]. By decreasing the expression of the M1 marker iNOS, the expression of the M2 marker CD206 increases, and the immune regulation of miR-451a promotes bone healing [28].

Based on the above research status of ADSC-Exos, some research results and the great prospects for clinical application, this article aimed to study the effectiveness of ADSC-Exos for the treatment of animal skin wounds and includes a meta-analysis of exosomes from general wounds and diabetic ulcer wounds in *in vitro* models of animals to provide a theoretical basis for clinical translation.

## Methods

### Protocol

The protocol for this study was established in accordance with the updated Preferred Reporting Items for Systematic Reviews and Meta-analyses (PRISMA) guidelines [29]. The study’s protocol was registered on PROSPERO (CRD42022369930, 22 November 2022).

### Literature search strategy

We searched PubMed, Cochrane, MEDLINE Complete, Web of Science databases, CNKI and Wanfang data for articles published without restriction on publication dates to 15 November 2022.The search terms used for the study were as follows: ‘adipose mesenchymal stem cell-derived exosomes’, ‘adipose-derived stem cell-derived exosomes’, ‘adipose-derived mesenchymal stem cells’, ‘ADSC-Exos', ‘ADMSCs-Exos’, ‘skin’, ‘wound’, ‘wounds’, ‘heal’, ‘healing’. The search was limited to animal trial studies. In addition, we performed a manual search of the references of the studies to obtain further potential studies.

### Outcome variables

#### Primary outcome variables

The primary outcome variable was the wound healing rate.

#### Secondary outcome variables

The secondary outcome variables were blood vessel density, collagen deposition, inflammatory markers, wound re-epithelization and the migration of skin fibroblasts.

### Study selection process

After removing duplicates, all identified citations were imported into Stata 17 for management. The search strategies used were peer-reviewed by two independent librarians and the full texts of any potentially relevant studies were screened to determine final eligibility. In cases of disagreement between reviewers, a consensus was achieved through discussion with a third team member. The study selection process was summarized using a flow diagram, as per PRISMA recommendations.

The inclusion criteria for studies were as follows: (1) experimental animal models; (2) experimental group receiving ADSC-Exo therapy; (3) control group receiving only nonfunctional solutions, vehicle or no treatment; (4) primary outcome of wound healing rate; and (5) secondary outcomes of blood vessel density, collagen deposition, inflammatory markers, wound re-epithelization and migration of human skin fibroblasts.

The exclusion criteria for studies were as follows: (1) had no control group in the study or had ADSC-Exos compared with another therapy; (2) was a case report, review or clinical trial; (3) lacked available data; and (4) had repeated publications.

### Data extraction

Relevant data were extracted by two independent reviewers from the included studies using a standardized and pilot-tested data extraction form in Excel (Microsoft, Seattle, WA, USA). In studies where the raw data were not presented, the data were extracted from figures using Digitizelt (version 2.2; Braunschweig, Germany). The following data were collected. (1) Study characteristics: authors, publication year and country of study. (2) Study populations: species, strain, diabetic model and wound size. (3) Intervention characteristics: ADSC dose and route of administration. (4) Study design: comparator, sample size and ADSC isolation and characterization methods. (5) Outcomes: wound healing rate, blood vessel density, collagen deposition, inflammatory marker levels, wound re-epithelization and migration of human skin fibroblasts. In addition, adverse events and details concerning the risk of bias were extracted.

### Literature quality evaluation

STAIR [30] was used to assess the quality of the included studies. The items were as follows: (1) Sample size calculation. (2) Inclusion and exclusion criteria. (3) Randomisation. (4) Allocation concealment. (5) Reporting of animals excluded from the analysis. (6) Patients were blinded to the assessment of outcome. (7) Reporting potential conflicts of interest and study funding. Rate “Yes”, “No” or “Unclear”.

### Statistical analysis

The data were analyzed by the system evaluation software Stata 17. All outcomes were regarded as continuous data and are presented as the standard mean difference (SMD) with 95% confidence intervals (CIs); *p* < 0.05 corresponded to the combined effect size, which indicated that there was a significant difference between the experimental group and the control group [31]. The Cochrane Q test and the *I^2^* test were applied to evaluate heterogeneity among the studies. A fixed-effects model was used to synthesize each outcome measure quantitatively. In the case of statistically significant between-study heterogeneity, random effects models were applied. The *I^2^* test was used to assess heterogeneity. A value >50% was considered to indicate substantial heterogeneity. Sensitivity analysis was performed using Stata 17 to compare the new combined effect size with the original combined effect size to determine whether there was any substantial change in the results. If the effect size corresponding to a study fell outside the 95% CIs, exclusion of the study had an impact on the total combined effect size. Second, if the combined effect size after a study is excluded significantly differs from the combined effect size without exclusion, the results are reversed; i.e. the difference changes, which indicates that the study has an impact on the total combined effect size.

## Results

### Study selection

**Figure 1:**
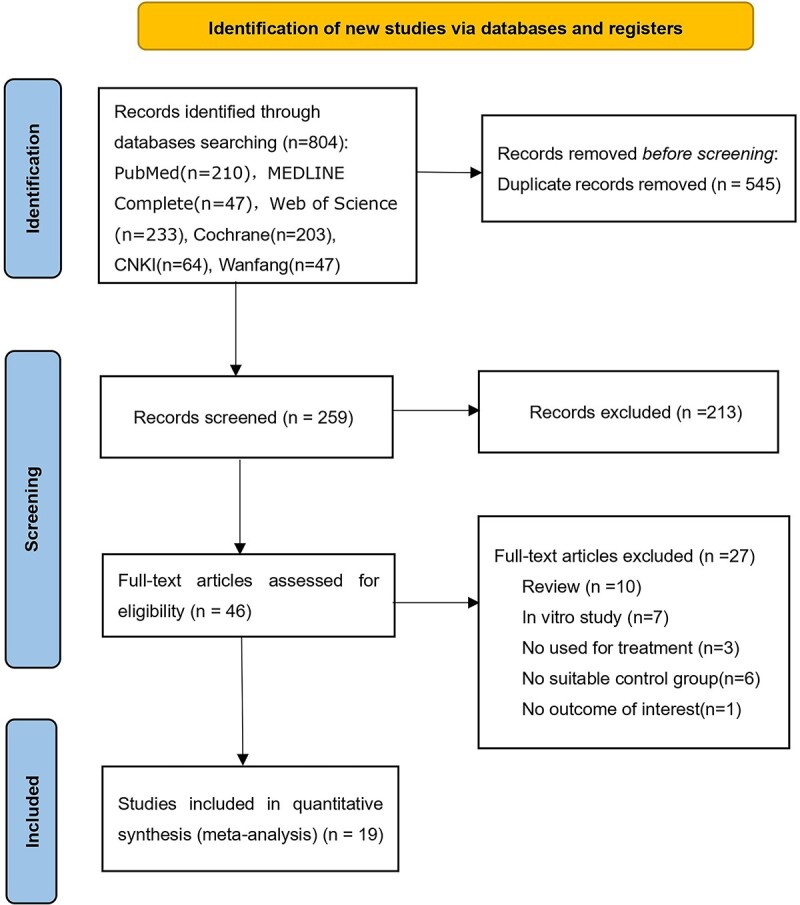
Flowchart of the study selection process

A total of 804 relevant records were retrieved, and after eliminating duplicate documents, 259 studies were retained. After preliminary reading of the titles and abstracts, 213 articles were excluded. We ultimately included 19 studies [32–50] (Figure 1).

### Study characteristics

The characteristics of the 19 included studies are summarized in Table 1. The study publication dates ranged from 2019 to 2022. Total sample size was 356. A total of 14 studies [32, 33, 37, 38, 40, 42–51] used mice and 5 studies [34–36, 39, 41] used rats. One study was in Iran [41], whereas the remaining 18 were conducted in China. Five studies [36, 41, 45, 46, 50] reported the use of allogeneic adipose tissue and 14 studies [32–35, 37–40, 42–44, 47–49] used xenogenic adipose tissue. The methods used in the present study for isolating and demonstrating the presence of exosomes included extracting and identifying exosomes via ultrahigh-speed centrifugation; observing the morphology of the ADSC-Exos via transmission electron microscopy; performing nanoparticle tracking analysis to determine the particle size distribution; and performing Western blotting to detect membrane surface signature proteins, including CD1, CD9, CD63, CD81 and CD82. All included studies used rodent models with full-layer skin defect wounds on the back or foot with a diameter of 4–20 mm. The control group included any type of control, including phosphate-buffered saline and the placebo.

**Table 1:** Characteristics of the included studies

**First author**	**Year**	**Country**	**Animal(number)**	**RCT**	**ADSCs source**	**Wound**	**Positive surface markers**		**Negative surface markers**		**Method**
**General wounds**							**Stem cell markers**		**Stem cell markers**	**Exosome markers**	
Cao *et al*. [32]	2020	China	BALB/c mice (5)	NA	Human adipose tissue	Diameter 10 mm	CD44, CD105	Not described	CD34, CD45	Not described	Subcutaneous injection; 100 μg/100 μl
Li, Q *et al*. [33]	2021	China	BALB/c mice (10)	NA	Human adipose tissue	10 × 10 mm	CD1, CD44, CD90	CD1, CD9, CD63	Not described	Not described	Subcutaneous injection; 200 μl
Li, C *et al*. [34]	2022	China	SD rats (3)	RCT	Human adipose tissue	8 × 8 mm	CD1, CD29, CD44, CD90	CD9, CD63	CD105	Not described	Subcutaneous injection and intradermal injection; 100 μg/1 μl
Liu-1 *et al.* [35]	2022	China	SD (5)	NA	Human adipose tissue	10 × 10 mm	Not described	Not described	Not described	Not described	Cover wound; NA
Liu-2 *et al.* [36]	2022	China	SD rats (6)	RCT	Mouse adipose tissue	Diameter 10 mm	Not described	Not described	Not described	Not described	Cover wound; 400 μl
Li, P *et al.* [37]	2022	China	C57BL/6 mice (6)	RCT	Human adipose tissue	10 × 10 mm	Not described	CD63, HSP70	Not described	Not described	Subcutaneous injection; 5 μg/100 μl
Lu *et al.* [38]	2020	China	Kunming mice (14)	NA	Human adipose tissue	Diameter 12 mm	CD105, CD44, CD73, CD90	Not described	CD14, CD19, CD34, CD45, HLA-DR	Not described	Subcutaneous injection; 200 μg/100 μl
Ma *et al.* [39]	2022	China	SD rats (15)	RCT	Human adipose tissue	Diameter 10 mm	CD90, CD105	CD63, CD81	CD34, CD45	Not described	Subcutaneous injection; 0.2 ml
Shen *et al.* [40]	2022	China	BALB/c mice (12)	RCT	Human adipose tissue	10 × 10 mm	CD29, CD44, CD73, CD90	CD9, CD63,TSG101	CD34, CD45	Not described	Subcutaneous injection; 1 mg/ml
Shilan *et al.* [41]	2020	Iran	Wistar rats (4)	NA	Rat adipose tissue	Diameter 15 mm	CD44, CD73, CD90	CD63	CD45	Not described	Cover wound; 300 μl
Yang *et al.* [42]	2020	China	BALB/c mice (6)	NA	Human adipose tissue	10 × 10 mm	CD29, CD44, CD90	Not described	CD34, CD45	Not described	Subcutaneous injection; 2 ml
Zhou *et al.* [43]	2022	China	ICR mice (6)	RCT	Human adipose tissue	15 × 15 mm	CD73, CD90, CD105	CD9, CD63, CD81	HLA-DR, CD45	Not described	Cover wound; 100 μg/100 μl
Zhu *et al.* [44]	2022	China	C57BL/6 mice (6)	RCT	Human adipose tissue	Diameter 20 mm	CD90, CD105	CD9, CD63, CD82	CD34, CD45	Not described	Subcutaneous injection; 200 μg
**Diabetic wounds**											
Shi-1 *et al*. [45]	2022	China	C57BL mice (6)	RCT	Mouse adipose tissue	Diameter 4 mm	CD90, CD29, CD105, CD44	CD63, CD81	CD34, vWF	Not described	Subcutaneous injection; 200 μg/100 μl
Shi-2 *et al.* [46]	2022	China	C57BL/6 mice (10)	RCT	Mouse adipose tissue	Diameter 4 mm	CD29, CD90, CD105, CD44	CD9, CD63, CD81	CD34, vWF	Not described	Subcutaneous injection; 200 μg/100 μl
Xiao *et al*. [47]	2021	China	BALB/c mice (10)	RCT	Human adipose tissue	Diameter 10 mm	Not described	CD9, CD81	Not described	Not described	Cover wound; 100 μg/100 μl
Wang-1 *et al*. [48]	2020	China	BALB/c mice (12)	RCT	Human adipose tissue	Diameter 8 mm	Not described	CD63	Not described	Not described	Subcutaneous injection; 0.2 ml
Wang-2 *et al.* [49]	2021	China	SCID mice (30)	RCT	Human adipose tissue	Diameter 8 mm	Not described	CD81, CD6, TSG101	Not described	Not described	Intraperitoneal injection; 200 μl
Wang *et al.* [50]	2019	China	ICR mice (12)	RCT	Mouse adipose tissue	Diameter 10 mm	Not described	Not described	Not described	Not described	NA; 100 μl

*NA* not available, *RCTs* randomised controlled trials, *ADSCs* adipose-derived stem cells

### Quality assessment

STAIR was used to assess the quality of the included articles. Most of those studies were randomised controlled trials (RCTs) but did not specify the randomisation allocation method used. Some articles neither described the use of randomisation nor mentioned allocation concealment. More details on the evaluation of study quality are provided in Table 2.

**Table 2:** Literature quality evaluation

**Study**	**A**	**B**	**C**	**D**	**E**	**F**	G
Cao *et al*. [32]	No	Unclear	Unclear	No	Unclear	Unclear	Yes
Li *et al*. [33]	No	Unclear	Unclear	No	Unclear	Unclear	Yes
Li *et al*. [34]	No	Unclear	Unclear	No	Unclear	Unclear	Yes
Liu *et al*. [35]	No	Unclear	Unclear	No	Unclear	Unclear	Yes
Liu *et al*. [36]	No	Unclear	Unclear	No	Unclear	Unclear	Yes
Pi *et al*. [37]	No	Unclear	Unclear	No	Unclear	Unclear	Yes
Lu *et al*. [38]	No	Unclear	Unclear	No	Unclear	Unclear	Yes
Ma *et al*. [39]	No	Unclear	Unclear	No	Unclear	Unclear	Yes
Shen *et al*. [40]	No	Yes	Yes	No	Unclear	Unclear	Yes
Shilan *et al*. [41]	No	Unclear	Unclear	No	Unclear	Unclear	Yes
Yang *et al*. [42]	No	Unclear	Unclear	No	Unclear	Unclear	Yes
Zhou *et al*. [43]	No	Unclear	Unclear	No	Unclear	Unclear	Yes
Zhu *et al*. [44]	No	Unclear	Unclear	No	Unclear	Unclear	Yes
Shi *et al*. [45]	No	Unclear	Unclear	No	Unclear	Unclear	Yes
Shi *et al*. [46]	No	Unclear	Unclear	No	Unclear	Unclear	Yes
Xiao *et al*. [47]	No	Unclear	Unclear	No	Unclear	Unclear	Yes
Wang *et al*. [48]	No	Unclear	Unclear	No	Unclear	Unclear	Yes
Wang *et al*. [49]	No	Unclear	Unclear	No	Unclear	Unclear	Yes
Wang *et al*. [50]	No	Unclear	Unclear	No	Unclear	Unclear	Yes

*A* Sample size calculation, *B* inclusion and exclusion criteria, *C* randomisation, *D* allocation concealment, *E* reporting of animals excluded from the analysis, *F* blinded assessment of outcome, *G* reporting potential conflicts of interest and study funding

### General wounds meta-analyses

#### Wound healing rate

**Figure 2:**
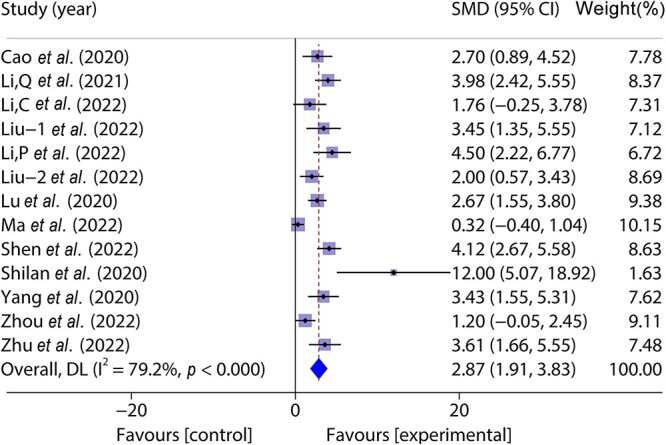
Forest plot shows the effect of wound healing on the 7th day of exosome treatment, and positive SMD indicates an increase in wound healing rate. *DL *Der simonian Laird Random effects method, *CI* confidence interval, *SMD* standard mean difference

A total of 13 studies [32–44] included 196 animal models and experimental (*n* = 98) and control (*n* = 98) groups and reported day 7 wound healing rates; heterogeneity existed between the studies (*p* < 0.05, *I^2^* > 50%) and a random effects model was used. Meta-analysis of the results showed that wound healing was faster in the experimental group than in the control group on the 7th day of the experiment (SMD = 2.87, 95%CI: 1.91–3.83, *p* < 0.001) (Figure 2).

**Figure 3:**
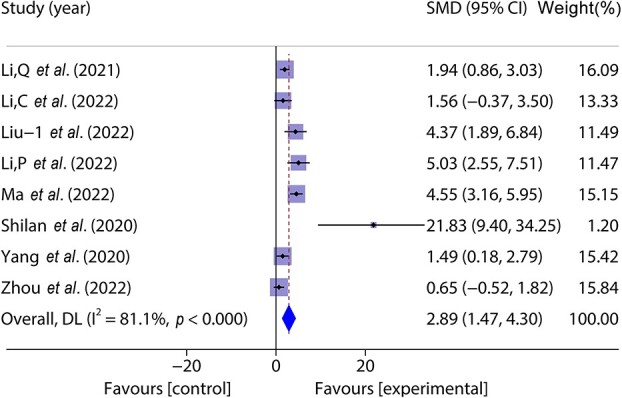
Forest plot shows the effect of wound healing on the 14th day of exosome treatment, and positive SMD indicates an increase in wound healing rate. *CI* confidence interval, *SMD* standard mean difference, *DL *Der simonian Laird Random effects method

Eight studies [33–35, 37, 39,41–43] included 110 animal models and experimental (*n* = 55) and control (*n* = 55) groups and reported day 14 wound healing rates; there was heterogeneity between the studies (*p* < 0.05, *I^2^* > 50%) and a random effects model was used. The result of the meta-analysis showed that wound healing was faster in the experimental group than in the control group on the 14th day of the experiment. (SMD = 2.89, 95%CI: 1.47–4.30, *p* < 0.001) (Figure 3).

#### Collagen deposition

**Figure 4:**
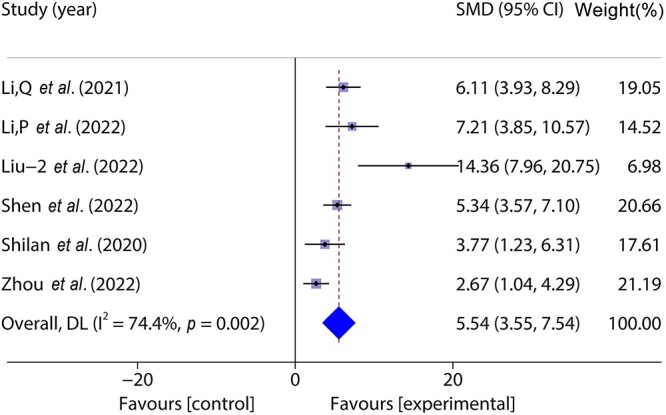
**F**orest plot shows the effect of the percentage of collagen deposition. *CI* Confidence interval, *SMD* standard mean difference, *DL* Der simonian Laird Random effects method

Six studies [33, 36, 37, 40, 41, 43] included 88 animal models and experimental (*n* = 44) and control (*n* = 44) groups and reported collagen deposition on wounds; heterogeneity existed between the studies (*p* < 0.05, *I^2^* > 50%) and a random effects model was used. The results of the meta-analysis showed that the percentage of collagen deposition in the experimental group was greater than that in the control group (SMD = 5.54, 95%CI: 3.55–7.54, *p* < 0.001) (Figure 4).

#### Blood vessel density

**Figure 5:**
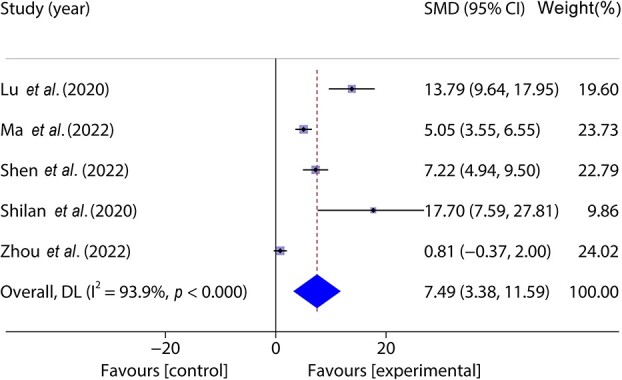
Forest plot shows the blood vessel density. *CI* confidence interval, *SMD* standard mean difference, *DL* Der simonian Laird Random effects method

Five studies [38–41, 43] included 102 animal models and experimental (*n* = 51) and control (*n* = 51) groups, and reported blood vessel density on wounds; there was heterogeneity between the studies (*p* < 0.05, *I^2^* > 50%) and a random effects model was used. The results of the meta-analysis showed that the blood vessel density in the experimental group was greater than that in the control group (SMD = 7.49, 95%CI: 3.38–11.59, *p* < 0.001) (Figure 5).

#### Wound re-epithelization

**Figure 6:**
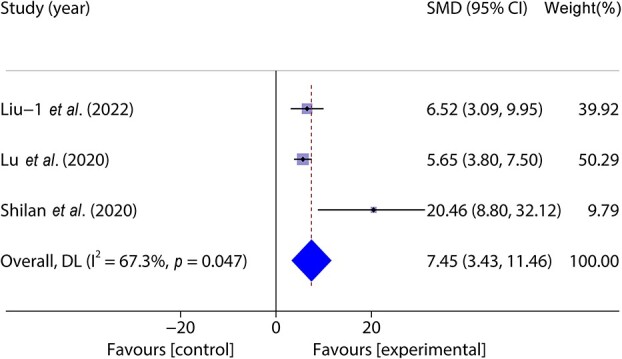
Forest plot shows the degree of re-epithelialization. *CI* confidence interval, *SMD* standard mean difference, DL Der simonian Laird Random effects method

Three studies [35, 38, 41] included 46 animal models and experimental (n = 23) and control (*n* = 23) groups and reported wound re-epithelialization; heterogeneity existed between the studies (*p* < 0.05, *I^2^* > 50%) and a random effects model was used. The results of the meta-analysis showed that the degree of re-epithelialization in the experimental group was greater than that in the control group (SMD = 7.45, 95%CI: 3.43–11.46, *p* < 0.001) (Figure 6).

#### Migration of skin fibroblasts

**Figure 7:**
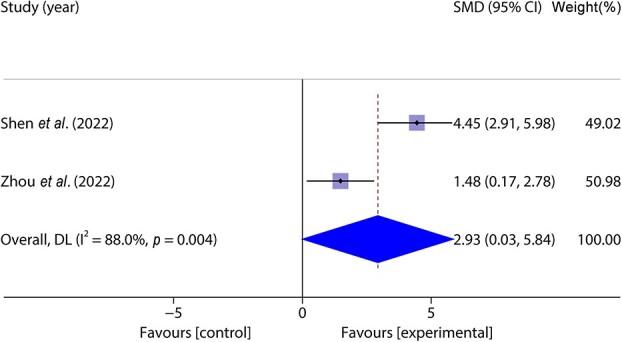
Forest plot shows the number of skin fibroblasts. *CI* confidence interval, *SMD* standard mean difference, *DL* Der simonian Laird Random effects method

Two studies [40, 43] included 36 animal models and experimental (*n* = 18) and control (*n* = 18) groups and reported the number of skin fibroblasts, with heterogeneity between studies (*p* < 0.05, *I^2^* > 50%), a random-effects model was used. The results of the meta-analysis showed that the number of migrating skin fibroblasts was greater in the experimental group than in the control group (SMD = 2.93, 95%CI: 0.03–5.84, *p* < 0.001) (Figure 7).

**Figure 8:**
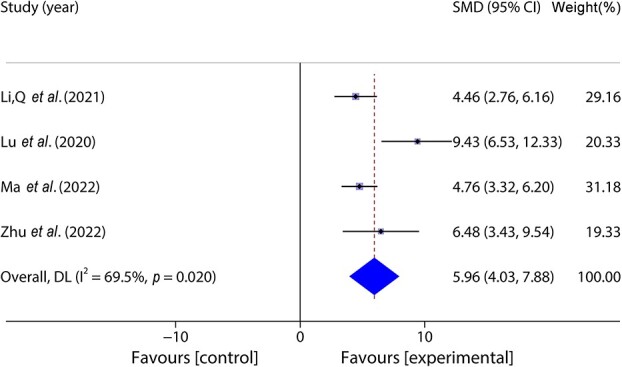
**F**orest plot shows the migration of skin fibroblasts. *CI* confidence interval, *SMD* standard mean difference, *DL* Der simonian Laird Random effects method

Four studies [33, 38, 39, 44] included 86 animal models and experimental (*n* = 43) and control (*n* = 43) groups and reported the migration of skin fibroblasts; heterogeneity existed between the studies (*p* < 0.05, *I^2^* > 50%) and a random effects model was used. The results of the meta-analysis showed that the percentage of fibroblasts was greater in the experimental group than in the control group (SMD = 5.96, 95%CI: 4.03–7.88, *p* < 0.001) (Figure 8).

### Diabetic wounds meta-analyses

#### Wound healing rate

**Figure 9:**
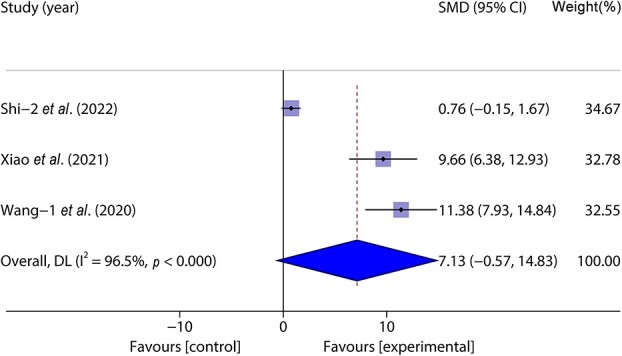
Forest plot shows the effect of wound healing on the 7th day of exosome wound treatment wound, and positive SMD indicates an increase in wound healing rate. *CI* confidence interval, *SMD* standard mean difference, *DL* Der simonian Laird Random effects method

Three studies [46–48] included 64 animal models and experimental (*n* = 32) and control (*n* = 32) groups and reported day wound healing rates; heterogeneity existed between the studies (*p* < 0.05, *I^2^* > 50%) and a random effects model was used. The results of the meta-analysis showed that there was no significant difference in the wound healing rate between the experimental group and the control group on the 7th day. (SMD = 7.13, 95%CI: −0.57–14.83, *p* > 0.05) (Figure 9).

**Figure 10:**
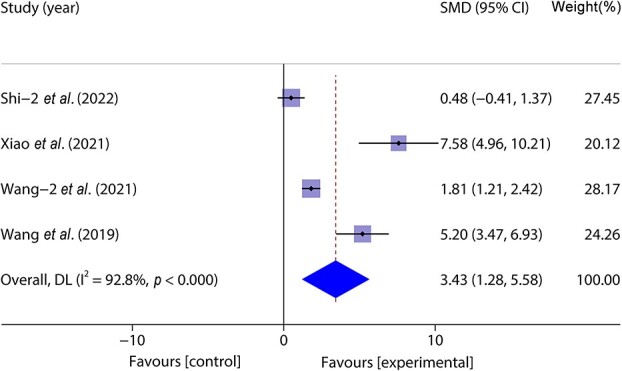
**F**orest plot shows the effect of wound healing on the 14th day of exosome wound treatment, and positive SMD indicates an increase in wound healing rate. *CI* confidence interval, *SMD* standard mean difference, *DL* Der simonian Laird Random effects method

Four studies [46, 47, 49, 50] included 124 animal models and experimental (*n* = 62) and control (*n* = 62) groups and reported day 14 wound healing rates; there was heterogeneity between the studies (*p* < 0.05, *I^2^* > 50%) and a random effects model was used. The results of the meta-analysis showed that wound healing was faster in the experimental group on the 14th day of the experiment (SMD = 3.43, 95%CI: 1.28–5.58, *p* < 0.001) (Figure 10).

#### Collagen deposition

**Figure 11:**
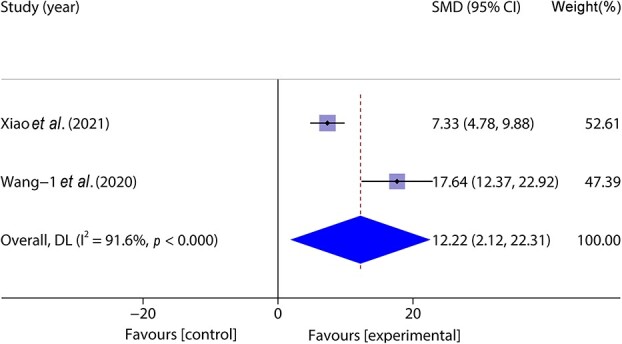
**F**orest plot shows the effect of the percentage of collagen deposition. *CI* confidence interval, *SMD* standard mean difference, *DL* Der simonian Laird Random effects method

Two studies [47, 48] included 44 animal models and experimental (*n* = 22) and control (*n* = 22) groups and reported collagen deposition on wounds; there was heterogeneity between the studies (*p* < 0.05, *I^2^* > 50%) and a random effects model was used. The results of the meta-analysis showed that the percentage of collagen deposition in the experimental group was greater than that in the control group. (SMD = 12.22, 95%CI: 2.12–22.31, *p* < 0.001) (Figure 11).

#### Blood vessel density

Three studies [45, 47, 48] included 196 animal models and experimental (*n* = 96) and control (*n* = 96) groups and reported blood vessel density on wounds. Meta-analysis revealed that there was no statistically significant difference in blood vessel density between the experimental and control groups (*p* = 0.662).

### Publication bias

**Figure 12:**
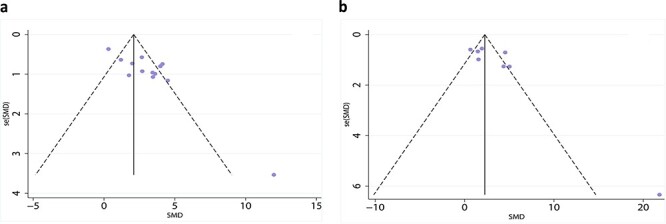
Funnel plot for wound healing rate on day 7 (**a**) and day 14 (**b**) in the general wounds group. *SMD *standard mean difference

A funnel plot was used to detect publication bias. In general wounds, the healing rate was measured on days 7 and 14, each point was concentrated at the top of the figure, indicating a small bias, but there was one potential missing study (Figure 12). Too few studies were included in the diabetic wound group to perform a funnel plot. Begg’s test was used to evaluate the wound healing rate in the general wound group on day 7 in 13 articles, *p* = 0.077 > 0.05. The wound healing rate on day 14 was reported in 8 articles, *p* = 0.108 > 0.05. The results suggest that no significant publication bias was detected. Too few studies were included in the diabetic wound group to perform Begg’s test.

### Sensitivity analysis

Sensitivity analysis of wound healing rate, collagen deposition, blood vessel density, wound re-epithelialization and fibroblast migration was performed for the general wound group on the 7th and 14th days. No effect size corresponding to one study was found to fall outside the 95% CI, and no reversal of the total combined effect size was found, indicating that no one study had an impact on the total combined effect size. The meta-analysis results for each outcome index were robust. Similarly, sensitivity analysis of the wound healing rate, collagen deposition and blood vessel density in the diabetic wound group on the 7th and 14th days revealed that the effect size corresponding to one study fell outside the 95% CI, and the total combined effect size did not reverse, indicating that no single study had an impact on the total combined effect size; moreover, the conclusions of the meta-analyses of each outcome indicator were also robust.

## Discussion

Diseases in which skin wounds do not heal or delay healing cause considerable psychological pressure and mental burden to patients. At present, wound repair treatment is the focus of clinical and scientific research, and this topic is an urgent problem to be solved. Stem cell exosome therapy has emerged as a treatment in recent years. Many experts and scholars have proven via animal tests and *in vitro* experiments that stem cell exosomes participate in wound repair in a variety of ways, such as by participating in wound cell proliferation and differentiation, promoting angiogenesis, regulating collagen synthesis, upregulating growth factors and regulating the inflammatory response [52–55]. Hence, stem cell exosomes have unlimited potential for wound healing. These conclusions have been verified, and positive results have been obtained in preclinical animal and cell studies; however, the feasibility of future clinical application needs further exploration and discovery. At present, ADSC-Exo extraction is efficient and safe during storage, the wound repair effect is remarkable, the evaluation is good and the advantages are obvious. However, skin wound repair is a complex and dynamic process that is especially involved in repairing various biological behaviors. Earlier studies have shown that ADSC-Exos have better potential than stem cell therapy. ADSC-Exos have shown satisfactory effects on wound repair in terms of regulating the inflammatory response, promoting epithelial cell and vascular regeneration, participating in cell proliferation and migration, and accelerating the wound healing process, which is consistent with the conclusions of our study. For diabetic patients, the high-glucose microenvironment formed by diabetes leads to changes in internal and external factors, such as abnormal immune function, increased oxidative stress, abnormal miRNA expression patterns and abnormal metabolism, resulting in increased levels of inflammatory markers and decreased functions of vascular endothelial cells and fibroblasts [56, 57]. As a result, the regeneration of wound granulation tissue is limited, the inflammatory period is prolonged and wound healing is delayed. In recent years, an increasing number of researchers have investigated the molecular mechanism through which ADSC-Exos repair ischemic tissue and promote angiogenesis to provide more advanced treatments for chronic and refractory wounds. To further improve the healing effect and clarify the efficacy and mechanism of adipose stem cell exosomes in skin wound repair, this review aimed to provide additional clinical evidence [12]. This article discusses the effect of ADSC-Exos on skin wound healing and evaluates the efficacy of these agents for the treatment of diabetic wounds.

**Figure 13:**
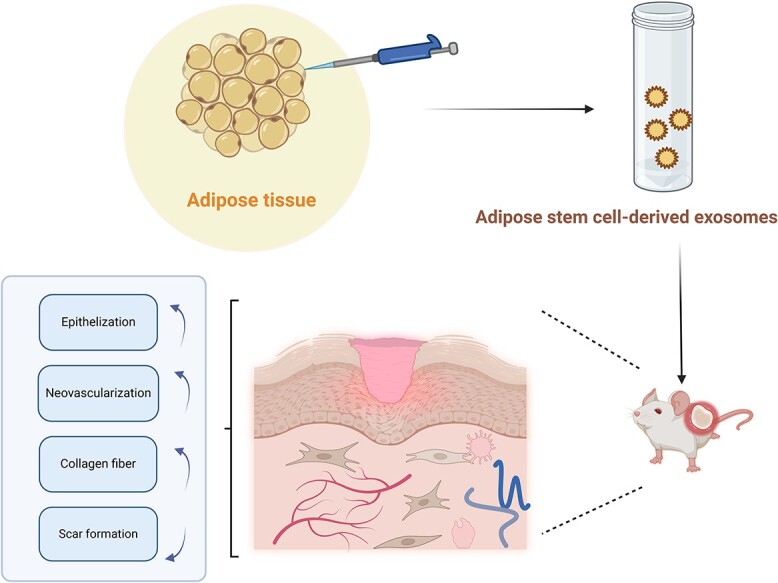
Adipose stem cell-derived exosomes (ADSC-Exos) promote healing. Figure created using BioRender (https://biorender.com/)

**Figure 14:**
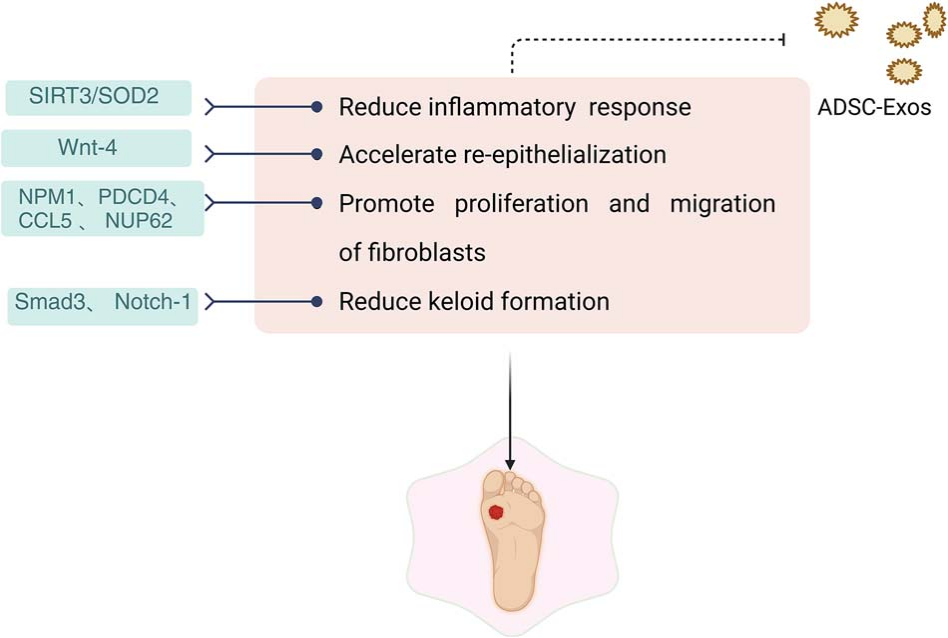
Role of ADSC Exos in wound healing. Figure created using BioRender (https://biorender.com/). *ADSC-Exos* adipose stem cell-derived exosomes

In our analysis, general wounds vs. diabetic wounds were chosen because the number of patients facing these two clinical problems is significant, and preclinical research models can guide the final clinical application and provide additional treatment strategies. We first selected the wound healing rate as the primary outcome and analyzed whether ADSC-Exos had an apparent effect on wound healing. Compared with those in the control group, the use of ADSC-Exos significantly improved the wound healing rate and accelerated wound healing in general and in diabetic patients. Secondary outcomes focused on the mechanisms affecting wound healing and tissue regeneration, as demonstrated in previous studies (Figures 13 and 14).

The results of this meta-analysis showed that, compared with those in the control group, the wounds in the general wound and diabetic wound intervention groups had greater wound healing rates and greater collagen synthesis on the 7th and 14th days after ADSC-Exo application, which proved that the combination treatment was effective at improving full-thickness skin wound healing and promoting collagen remodeling. The increased density of blood vessels indicates that ADSC-Exos can promote wound neovascularization, which provides a new idea for the treatment of ischemia–reperfusion injury, insufficient neovascularization of flaps in artificial skin and poor healing. Increased re-epithelialization and fibroblast proliferation and migration can promote skin wound healing. According to the results of the present meta-analysis, ADSC-Exos significantly improved the wound healing rate in both general and diabetic wounds, and these findings are also supported by the findings of other studies. However, additional RCTs are needed to verify this issue in other ischemic wound studies.

In addition, the intervention group also exhibited positive results in terms of inhibiting the gene expression and protein secretion of inflammatory factors, such as tumour necrosis factor-alpha (TNF-α), interleukin (IL)-6 and IL-1β, suggesting that ADSC-Exos inhibited the inflammatory response and regulated immune function by regulating signaling pathways; however, only one study [40]has described inflammatory factors. According to another study, human adipose MSC-derived exosomes are also involved in the immune regulation of T cells [58]. Heo [59] reported that the protein levels of TNF-α, IL-6 and IL-8 decreased and that the concentration of IL-10 increased significantly. There were few measurements of inflammatory factors in the included studies, and additional animal studies are needed to explain this phenomenon. Further research is needed on immune regulation.

In recent years, there has been increasing research on stem cell exosomes. ADSC-Exos play a role in wound healing and repair through different pathways. In burn wounds, stem cell-derived exosomes can inhibit the inflammatory signaling pathway, reduce inflammation, and promote the proliferation and migration of fibroblasts [60]. In chronic diabetic wounds, Zhang [61] reported that ADSC-Exos could reduce the inflammatory response by regulating SIRT3/SOD2, and some functional miRNAs can be transported through ADSC-Exos via Wnt/β-catenin signaling [62]. Topical application of exosomes containing MALAT1 in ischemic wounds can promote wound angiogenesis [63]. ADSC-Exos promote collagen deposition through the PI3K/Akt signaling pathway to further accelerate wound healing [53]. AdMSC-Exos can reduce keloid formation by inhibiting the protein expression of Smad3 and Notch-1 [64]. ASC exosomes contain miRNAs, which can inhibit NPM1, PDCD4, CCL5 and NUP62 and stimulate the proliferation of fibroblasts [65]. Studies have shown that ADSC-Exos containing miR-451a can influence the expression of inflammatory cytokines by regulating M1/M2 macrophage polarization [66, 67]. Several studies have shown that hypoxic preconditioned ADSC-Exos significantly enhance angiogenesis through VEGF/VEGF-R and increase the number of CD31-positive cells [68–70]. In rats, activation of Wnt4 promoted β-catenin nuclear translocation, and activity accelerated wound re-epithelialization [71]. Based on these findings, we concluded that ADSC-Exos are involved in the regulation of different wound repair periods and can have a more positive effect on promoting skin healing than can the control treatment. Wound repair often involves a variety of cellular mechanisms. To a certain extent, this study revealed that wound repair depends not only on the regulation of the proliferative and remodeling stages of skin healing but also on the inflammatory stage. In addition to the outcome indicators included in this meta-analysis, such as collagen deposition, neovascularization and re-epithelialization, there may be other factors that influence the effectiveness of ADSC-Exos in wound repair. These results also support the findings of previous studies and suggest that ADSC-Exo therapy has a positive effect on promoting diabetic wound repair.

During the study, we found that the combination of ADSC-Exos with new materials had more exciting effects than the other agents, among which the most commonly used was hydrogel, which is organized by a 3D hydrophilic polymer network [72, 73]. As a new biological material, ADSC-Exos can be combined with other polymers to make wound dressings with antibacterial and antioxidant properties [43, 74]. Good gas and liquid semipermeability is required for skin wounds. Hydrogels containing ADSC-Exos, which act as slow-release scaffolds for exosomes, provide microenvironment protection for ADSC-Exos, improve the utilization rate of exosomes and promote wound healing [75]. ADSC-Exos combined with hydrogels have great potential for treating wounds. At present, most of these studies involve animal models and further clinical studies are needed to verify these findings.

In the pre-clinical model, ADSC-Exos, as a cell-free therapy, participate in the change of the cellular microenvironment, and can activate molecular signaling pathways to promote skin tissue repair after hypoxia, hyperglycemia and other pretreatments. Clinical studies on exosomes derived from MSCs focus on diabetic wound healing, inflammation regulation, brain injury treatment, liver disease treatment, cardiovascular disease treatment, bone regeneration, novel coronavirus pneumonia treatment etc. However, standardized protocols for sustainable exosome production need to be formulated. Currently, most clinical studies on exosomes are still in the early stage of development. There is no detailed scientific research data to guide the clinic.

In this article, we summarize the general and diabetic wounds caused by full-thickness skin defects, and ADSC-Exo-mediated repair of these wounds have indeed been shown to be effective. However, many studies have reported irregularities and nonstandard treatments, resulting in some studies not meeting research standards and increasing statistical difficulties by having incomplete data reporting and no explanation of the use of randomisation. In addition, problems are encountered in statistics. In terms of experimental design, stem cells and exosomes are not standardized, the dosage of exosomes is not standardized—the volume alone cannot reflect the real dosage and more quantifiable indicators, such as protein quantity and even particle number, can be used—and the wound model is not standard. To better obtain authentic and reliable evidence, future *in vivo* or *in vitro* experiments should be more rigorous and standardized in design, produce more powerful evidence for preclinical research on exosomes and promote the clinical treatment and application of exosomes.

### Limitations

Our study has several limitations. First, some of the studies did not include RCTs, some included studies had a moderate–high risk of bias, the number of studies available for meta-analysis calculations was limited, and the number of articles was small, which was not enough to evaluate publication bias. Second, the experimental animals included in the study were all mouse or rat models, and large-animal experimental models were lacking. Therefore, large animal experiments are needed to validate these findings. Finally, the use of Digitizer software during data extraction may have resulted in some deviation of the data.

## Conclusions

As our data analysis indicated, ADSC-Exos performed well *in vitro* at repairing wounds, and wound healing was related to multiple mechanisms. ADSC-Exo therapy applied to general and diabetic wounds can promote neovascularization, improve epithelization and collagen fiber deposition, promote healing and reduce scar formation. Although the effectiveness of ADSC-Exos in wound repair has been demonstrated in animal studies, decision-making prospective trials based on ADSC-Exos are still needed before clear clinical recommendations can be provided to guide wound management.

## Abbreviations

ADSC-Exos: Adipose stem cell-derived exosomes; CI: Confidence interval; miRNA: MicroRNA; MSC: Mesenchymal stem cell; RCT: Randomised control trial; SMD: Standard mean difference.

## Funding

This work was in part supported by grants from the National Natural Science Foundation of China (No. 82272261) and Shanxi Province Foundation of China (No. 2022JC-58, No. 2021SF-341).

## Authors’ contributions

Jing-tao Wei (Data curation, Formal analysis, Investigation, Methodology, Software, Writing—original draft), Ting He (Data curation, Formal analysis, Writing—review & editing), Kuo Shen (Data curation, Software visualization, Writing—review & editing), Zhi-gang Xu (Data curation, Project administration, Visualization, Writing—review & editing), Jun-tao Han (Ideas resources, Supervision, Validation, Project administration, Writing—review & editing), and Xue-kang Yang (Ideas conceptualization, Funding acquisition, Methodology, Project administration, Writing—review & editing).

## Conflict of interest

The authors have no other relevant affiliations or financial involvement with any organization or entity with a financial interest in or financial conflict with the subject matter or materials discussed in the manuscript apart from those disclosed in the Funding section.

## Supplementary Material

AJE_Editing_Certificate_tkae025

Supplementary_Search_strategy_tkae025
