# Supplementary material for: Adipose stem cell-derived exosomes in the treatment of wound healing in preclinical animal models: a meta-analysis
Source: Burns Trauma. 2024 Aug 4;12:tkae025. doi: 10.1093/burnst/tkae025 (PMC11298109; doi:10.1093/burnst/tkae025)
Supplement: Supplementary_Search_strategy_tkae025 [file supplementary_search_strategy_tkae025.docx]

**Search strategy**

**Database：Pubmed**

Data searched: November 15, 2022

Records retrieved:**210**

((adipose mesenchymal stem cell-derived exosomes[Title/Abstract]) OR (adipose-derived stem cell-derived exosomes[Title/Abstract]) OR (adipose-derived mesenchymal stem cells[Title/Abstract]) OR (ADSC-Exos[Title/Abstract]) OR (ADMSCs-Exos[Title/Abstract])) AND ((wound[Title/Abstract]) OR (wounds[Title/Abstract]) OR (wounds heal[Title/Abstract]) OR (wound healing[Title/Abstract]) OR (skin[Title/Abstract]) OR (skin wound healing[Title/Abstract]) OR (skin wounds[Title/Abstract]))

**Database：Web of Science**

Data searched: November 15, 2022

Records retrieved: **233**

((((((TI=(adipose mesenchymal stem cell-derived exosomes)) OR TI=(adipose-derived mesenchymal stem cells)) OR TI=(adipose-derived stem cell-derived exosomes)) OR TI=(ADSC-Exos)) OR TI=(ADMSCs-Exos))) AND TS=(skin OR wound OR wounds OR heal OR healing OR wound healing OR skin heal OR wounds heal)

**Database：MEDLINE Complete**

Data searched: November 15, 2022

Records retrieved: **47**

(adipose mesenchymal stem cell-derived exosomes (Topic) or adipose-derived mesenchymal stem cells (Topic) or adipose-derived stem cell-derived exosomes (Topic) or ADSC-Exos (Topic) or ADMSCs-Exos (Topic)) and (wound healing (Topic) or skin heal (Topic) or wounds heal (Topic))

**Database：Cochrane**

Data searched: November 15, 2022

Records retrieved: **203**

(adipose mesenchymal stem cell-derived exosomes):ti,ab,kw OR (adipose-derived mesenchymal stem cells):ti,ab,kw OR (adipose-derived stem cell-derived exosomes):ti,ab,kw OR (ADSC-Exos OR ADMSCs-Exos):ti,ab,kw AND (skin OR wound OR wounds OR heal OR healing OR wound healing OR skin heal OR wounds heal):ti,ab,kw

**Database：CNKI**

Data searched: November 15, 2022

Records retrieved:**64**

Title:(adipose mesenchymal stem cell-derived exosomes) or Title:(adipose-derived mesenchymal stem cells) or Title:(adipose-derived stem cell-derived exosomes) or Title:(ADSC-Exos) or Title:(ADMSCs-Exos) and Title:(wounds heal) or Title:(skin heal) or Title:(wound healing)

**Database：Wanfang**

Data searched: November 15, 2022

Records retrieved:**47**

Title or keyword:(adipose mesenchymal stem cell-derived exosomes) or Title or keyword:(adipose-derived mesenchymal stem cells) or Title or keyword:(adipose-derived stem cell-derived exosomes) or Title or keyword:(ADSC-Exos) or Title or keyword:(ADMSCs-Exos) and Title or keyword:(wounds heal) or Title or keyword:(skin heal) or Title or keyword:(wound healing)
